# Impact of COVID-19 on Timing of Hip-Fracture Surgeries: An Interrupted Time-Series Analysis of the Pre/Post-Quarantine Period in Northern Italy

**DOI:** 10.34172/ijhpm.2021.103

**Published:** 2021-09-01

**Authors:** Jacopo Lenzi, Stefano Rousset, Maria Pia Fantini, Maria Michela Gianino

**Affiliations:** ^1^Department of Biomedical and Neuromotor Sciences, Alma Mater Studiorum – Università di Bologna, Bologna, Italy.; ^2^Department of Public Health Sciences and Paediatrics, Università di Torino, Torino, Italy.

**Keywords:** Quality of Care, Hip Fractures, Elderly, COVID-19 Pandemic, Interrupted Time Series, Italy

## Abstract

**Background:** To assess whether the imposition of the coronavirus disease 2019 (COVID-19) national quarantine (March 10, 2020) resulted in a shift in the proportion of patients operated for hip fracture on the day of admission, the following day and two days after admission in the region of Piedmont, northern Italy.

**Methods:** Interrupted time-series analysis (ITSA) comparing hospitalization rate and timing of hip-fracture surgeries between pre- and post-quarantine period. The same data observed in Piedmont the year before were included as a control time series with no "intervention" (quarantine) in the middle of the observation period.

**Results:** We found that 70.3% and 69.4% of hip-fracture patients received surgery within 2 days of hospital admission in the 16 weeks before and after the national quarantine, respectively. One-day surgery went from 46.0% to 46.5%, and same-day surgery from 13.3% to 12.4%. Unchanged trends were confirmed by ITSA after controlling for the 32-week time-series observed the year before. In the second week of March 2020, there was a borderline significant decrease in weekly hospital admissions for hip fractures as compared with that of the same week of March 2019 (–1.95 per 100 000, 95% CI = –4.10 to 0.21, *P* value =.075), followed by a weekly significant increase in the hospitalization rate (+0.14 per 100 000, 95% CI = 0.01 to 0.27, *P* value =.039), although the difference-in-differences of slopes failed to achieve statistical significance (0.19 per 100 000, 95% CI = –0.03 to 0.41, *P* value =.090).

**Conclusion:** Our study shows that the timing of hip-fracture surgery was unchanged during the lockdown period. This suggests that the healthcare systems can be resilient and able to guarantee a high-quality and safe healthcare to hip-fracture patients, even in the most challenging working conditions.

## Background

**Key Messages**

** Implications for policy makers**

- Indicators of the quality of care, such as timely hip-fracture surgery, are useful to monitor the response of healthcare systems during an emergency.
- During the coronavirus disease 2019 (COVID-19) pandemic, healthcare systems all around the world had to get reorganized to guarantee adequate care to all patients. This study suggests that it is possible to guarantee a high-quality and safe healthcare to hip-fracture patients, even during a pandemic.
- Our study brings to policy-makers’ attention the idea that quality measurement is essential, especially in time of crisis. Improving data quality can significantly help clinicians strengthen care delivery in this difficult time and avoid a decrease of healthcare systems performances.
- Our study suggests that resilience could play as an emerging property of healthcare systems when facing health emergencies. Monitoring quality of care indicators is important to understand how to improve healthcare systems resilience, in order to be adequately prepared for future threats.

** Implications for the public**

This study shows that, even during a global emergency, patients with hip fracture received high-quality care, with no difference compared with the pre-pandemic period. We think that this is an important information for public, as it demonstrates how our healthcare systems displayed resilience and ability to get reorganized. Our study is significant also for international audiences, as it gives an insight on how our healthcare system, one of the first stricken by coronavirus disease 2019 (COVID-19) outside of China, faced the emergency without forgetting non-COVID-19 patients needing acute surgery treatments. As this pandemic is a global emergency, affecting everyone, the public needs to know that quality of care can be guaranteed, at least for acute conditions, even during these difficult times.

Hip fractures are very common, especially among elderly patients. In Europe, the highest incidence rate is found in Sweden and Norway (920/100 000 in women and 399.3/100 000 in men) and the lowest in Switzerland and France (346/100 000 in women and 137.8/100 000 in men).^1^ The main risk factors are osteoporosis, older age, female sex and race.^2^ The epidemiological transition, with the population becoming increasingly older, is particularly evident in Italy, where age-related diseases, such as hip fractures, constitute a serious public health issue.^3,4^

 Since non-surgical treatment has several limitations, including prolonged immobilization and poor return to functional mobility, and is associated with complications such as decubitus ulcers, thromboembolic disease, pneumonias, urinary tract infections and mortality, reparative surgery is strongly recommended.^5,6^ A strong body of evidence has emerged in the last years showing that the ideal timing for reparative surgery should not exceed two days of initial presentation, in order to reduce mortality and improve quality of life.^7-9^ Many European countries, including Italy, acknowledged this evidence in clinical guidelines,^10^ and surgery timing is recognized as a significant indicator of quality of care for patients with hip fracture.^11^

 Since the outbreak of the coronavirus disease 2019 (COVID-19) pandemic in February 2020, Italy’s healthcare system has been put under enormous pressure, being forced to switch the vast majority of routine activity to the management of the emergency, thus determining a drop in the quality of care delivered to non-COVID-19 patients.^12^ Some hospitals suspended non-urgent outpatient orthopedic activity and elective surgery and, with the goal of maintaining a high quality of care and guaranteeing orthopedic trauma surgery that could not be postponed or delayed, several aspects of hospital organization and bed management were changed.^13^

 However, to our knowledge, there is still scarcity of large-scale data on the impact of the COVID-19 pandemic and the resulting stay-at-home measures on the quality of care delivered to elderly orthopedic patients. Piedmont, a region of northwestern Italy with 4.4 million inhabitants that covers nearly 25 400 km^2^, might serve as an epitome to conduct such investigation, because it is one of the first areas of the world that faced the pandemic outside China and, together with the other regions of Italy, has a high degree of local autonomy in managing healthcare delivery. As such, the aim of this study was to assess whether the imposition of the COVID-19 national quarantine on the second week of March 2020 resulted in a shift in the percentage of elderly patients who received timely hip-fracture surgery in Piedmont compared with that of the pre-quarantine period.

## Methods

 We collected the hospital discharge records (HDRs) of all patients admitted to the hospitals of Piedmont with a principal or secondary diagnosis of upper femur fracture (ICD-9-CM code 820). Data extraction was carried out utilizing anonymized data from the Regional Public Health Observatory (SEPI), Local Healthcare Authority TO3, Via Sabaudia 164, Grugliasco, TO 10095.

 In keeping with the specification of the indicator adopted by the *Programma Nazionale Esiti* (https://pne.agenas.it/index.php?lang=EN), HDRs were excluded from the analysis if any of the following criteria was met:

- Non-urgent hospital admission;
- Daytime hospital care, known in Italy as “day hospital admission,” which consists in a one-day admission to the hospital without overnight stay to perform diagnostic procedures and/or surgical, therapeutic or rehabilitative care^14^;
- Transfer from other hospital;
- Age <65;
- Polytrauma (diagnosis-related group 484–487);
- Diagnosis or medical history of malignant tumors (principal/secondary ICD-9-CM code 140.0–208.9, 238.6, V10);
- Death within one day of hospital admission and no surgery to repair hip fracture;
- Admission to a spinal injury unit, rehabilitation hospital or long-term care facility.

Hospitalization rates were obtained as the number of hospital admissions for hip fracture in the resident population aged ≥65 years per 100 000 inhabitants. Population data were retrieved from the Italian National Institute of Statistics (https://demo.istat.it/).

 Timely hip-fracture surgery among the cases described above was defined as any of the following procedures initiated within two calendar days of admission to the hospital: closed reduction of fracture with internal fixation (ICD-9-CM codes 79.10, 79.15); open reduction of fracture with internal fixation (79.30, 79.35); total or partial hip replacement (81.51, 81.52). We also investigated the percentage of cases surgically treated the next day (day 1) and on the same day as hospital admission (day 0).

 Hospital admission rates were standardized by sex and age (<80, 80–84, 85–89, ≥90 years) with direct standardization to Italy’s 2020 elderly population. Percentages of surgery were standardized by sex, age and enhanced Charlson index score (0, 1, ≥2),^15^ with direct standardization to the overall population of hip fractures observed in Piedmont over the study period.

 For descriptive purposes, we also gathered some characteristics of the admitting hospitals; more specifically, we collected hospital type/ownership, hospital location, and average annual caseload of hip fractures.

###  Statistical Analysis

 We performed an interrupted time-series analysis (ITSA), a quasi-experimental design which represents a desirable alternative to randomized studies when the latter are not feasible.^16^ Post-quarantine data were collected from March 11, 2020 to June 30, 2020 (16 weeks), while pre-quarantine data were collected from November 20, 2019 to March 10, 2020 (16 weeks). To reduce any source of confounding, the same data observed in Piedmont the year before, ie, between November 20, 2018 and July 1, 2019 (32 weeks), were included as a control time series with no intervention (quarantine) in the middle of the observation period.

 A two-group ITSA regression model assumes the following generic form:

*Y*_t_ *= β*_0_ *+ β*_1_ *T*_t_ *+ β*_2_ *X*_t_ *+ β*_3_ *X*_t_ *T*_t_ *+ β*_4_ *Z + β*_5_ *ZT*_t_ *+ β*_6_ *ZX*_t_ *+ β*_7_ *ZX*_t_ *T*_t_ *+ ϵ*_t_

 Where *Y*_t_ is an aggregated outcome variable measured at each time point *t* (in our study, weekly hospitalization and surgery rates), *T*_t_ is time since the start of the study, *X*_t_ is a dummy (indicator) variable representing the intervention (pre = 0, post = 1), *Z* is a dummy variable to denote the cohort assignment (study or control), and *ϵ*_t_ is the random error term. Here is the interpretation of the seven parameters that constitute the linear model:

- *β*_0_ = intercept (starting level) of the outcome variable in the control group;
- *β*_1 _= slope (trajectory) of the outcome in the control group until the introduction of the intervention;
- *β*_2 _= change in the level of the outcome that occurs in the period immediately following the introduction of the intervention in the control group;
- *β*_3_ = difference between pre-intervention and post-intervention slopes of the outcome in the control group;
- *β*_4_ = difference in the level between study and control prior to intervention;
- *β*_5_ = difference in the slope between study and control prior to intervention;
- *β*_6 _= difference-in-differences of the change of level between study and control;
- *β*_7 _= difference-in-differences of slopes between study and control.

As anticipated by the definitions of* β*_6_ and *β*_7_, causal inference from two-group ITSA is provided using the difference-in-differences approach, in which between-period changes in a study cohort are compared with changes in a control cohort over a similar timeframe (pre-post with-without). The two parameters *β*_4_ and *β*_5_ are useful to establish whether the study and control series are balanced on the level and the trajectory of the outcome variable in the pre-intervention period; if significantly different from 0, conclusions drawn from *β*_6_ and *β*_7_ might be biased. A visual exemplification of ITSA is provided in Linden and Arbor.^17^

 We computed robust (heteroscedasticity-consistent) standard errors to make valid inference about the regression coefficients. According to the Cumby–Huizinga test,^18^ there was no evidence of autocorrelation at any lag order.

 In keeping with the specification of the indicator adopted by the Organization for Economic Co-operation and Development (OECD), a sensitivity ITSA was performed on hip-fracture surgery after excluding HDRs with a diagnosis of upper femur fracture in secondary position. All data were analyzed using Stata version 15 (StataCorp. 2017. *Stata Statistical Software: Release 15.* College Station, TX: StataCorp LP).^17^ The significance level was set at 5%, and all tests were two-sided.

## Results

 In the region of Piedmont, we observed 2151 hospitalizations for hip fracture in the 16 weeks preceding the imposition of the national quarantine (sex- and age-standardized rate = 188.5 per 100 000), and 1722 in the following 16 weeks (sex- and age-standardized rate = 150.9 per 100 000). We registered an increased concentration of admissions to research and teaching hospitals (23.3% to 29.8%), combined with a decrease in the relative number of admissions to the hospitals of local healthcare authorities (74.5% to 68.9%) and to private facilities (2.1% to 1.3%). A summary of patient and hospital characteristics before and after the quarantine, including 2018/2019 control data, is provided in Table S1 (Supplementary file 1).

**Figure 1:**
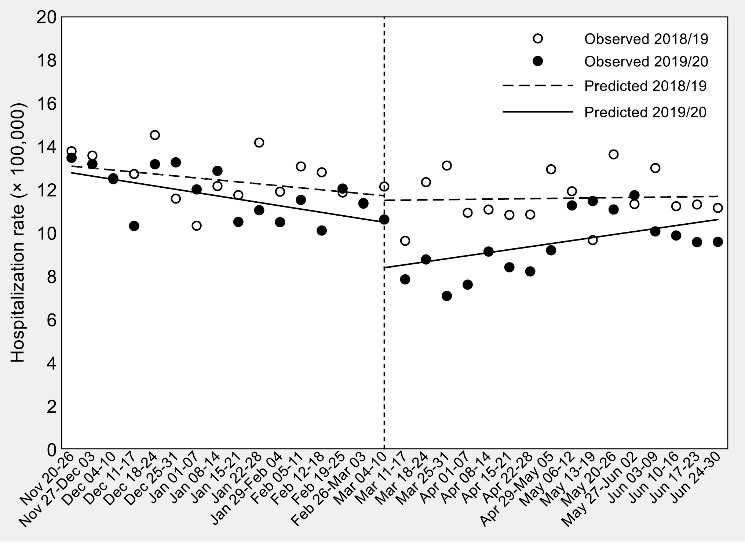
ITSA of Weekly Sex- and Age-Standardized Hip-Fracture Hospitalization Rates in Piedmont in the 16 Weeks Before and After Italy’s COVID-19 National Quarantine (Dashed Vertical Line); data observed the year before (2018/2019) are used for comparison.Abbreviations: ITSA, interrupted time-series analysis; COVID-19, coronavirus disease 2019. *Note:* The last day of the control period is July 1, because 2019 is a common (non-leap) year.

 Results of the ITSA on hip-fracture hospitalization rates are presented in Table 1 and Figure 1.

**Table 1:** Regression Table of ITSA on Weekly Sex- and Age-Standardized Hip-Fracture Hospitalization Rates in Piedmont Before and After Italy’s COVID-19 National Quarantine

**Variable**	**Coefficient (95% CI)**	***P *** **Value**
Intercept	13.17 (12.28, 14.06)	<.001
*T*_t_	–0.09 (–0.18, –0.01)	.038
*X*_t_	–0.30 (–1.69, 1.08)	.661
*X*_t_ *T*_t_	0.10 (–0.04, 0.24)	.143
*Z*	–0.25 (–1.57, 1.07)	.710
*ZT*_t_	–0.06 (–0.20, 0.08)	.379
*ZX*_t_	–1.95 (–4.10, 0.21)	.075
*ZX*_t_ *T*_t_	0.19 (–0.03, 0.41)	.090

Abbreviations: ITSA, interrupted time-series analysis; CI, confidence interval; COVID-19, coronavirus disease 2019. *Notes:* Data observed the year before (2018/2019) are used for comparison. *T*_t_ is time since the start of the study (November 20), *X*_t_ is an indicator variable that equals 1 in the weeks 11 to 26 of the tropical year (March 11, 2020/March 12, 2019 to June 30, 2020/July 1, 2019), and *Z* is an indicator variable that equals 1 in the “experimental” time series (November 20, 2019 to June 30, 2020). The post-quarantine trend between March 11, 2020 and June 30, 2020 can be obtained as *β*(*T*_t_) + *β*(*ZT*_t_) + *β*(*X*_t_*T*_t_) + *β*(*ZX*_t_*T*_t_) = –0.09 – 0.06 + 0.10 + 0.19 = +0.14.

 In the second week of March 2020, there appeared to be a decrease in weekly hospital admissions for hip fracture as compared with that observed in the same week of March 2019 (*β*_6 _= [8.39–10.64] – [11.50–11.81] = –1.95 per 100 000), although the difference-in-differences of the change of level failed to achieve statistical significance (95% CI = –4.10 to 0.21, *P *value = .075). The drop in the number of hospital admissions was followed by a weekly significant increase in the hospitalization rate (+0.14 per 100 000, 95% CI = 0.01 to 0.27, *P *value = .039) but, again, the difference-in-differences of slopes failed to achieve statistical significance (*β*_7 _= [0.14+0.15] – [0.01+0.09] = 0.19 per 100 000, 95% CI = –0.03 to 0.41, *P *value = .090).

**Figure 2:**
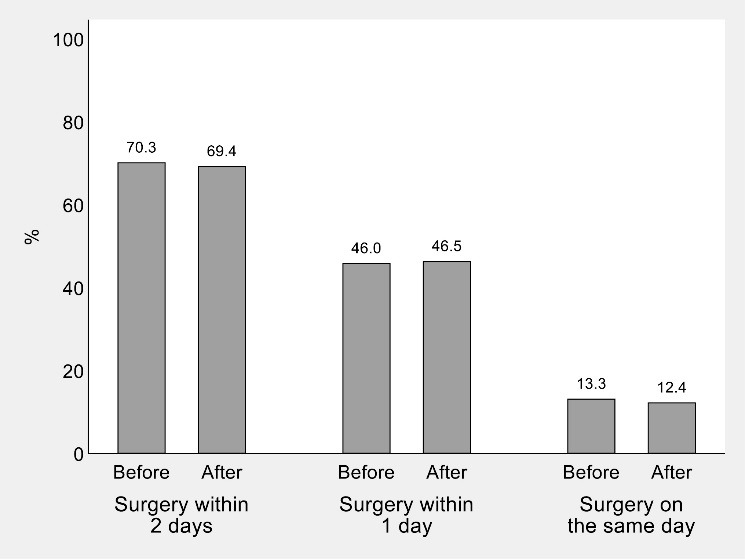
Sex-, Age- and Comorbidity-Standardized Percentages of Hip-Fracture Surgery Initiated Within 2 Days, Within 1 Day and on the Same Day as Hospital Admission in Piedmont in the 16 Weeks Before and After Italy’s COVID-19 National Quarantine.Abbreviation: COVID-19, coronavirus disease 2019. *Note:* No significant differences were found between the pre- and post-quarantine period on multivariable logistic regression analysis (two days: *P *value =.581; one day: *P *value =.731; same day: *P *value =.471).

**Figure 3:**
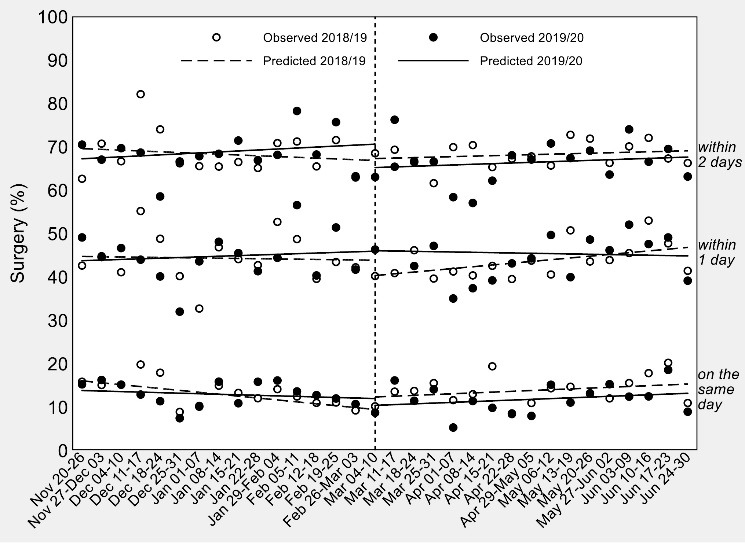
ITSA of Weekly Sex-, Age- and Comorbidity-Standardized Percentages of Hip-Fracture Surgery in Piedmont in the 16 weeks Before and After Italy’s COVID-19 National Quarantine (Dashed Vertical Line); data observed the year before (2018/2019) are used for comparison.Abbreviations: ITSA, interrupted time-series analysis; COVID-19, coronavirus disease 2019. *Note:* The last day of the control period is July 1, because 2019 is a common (non-leap) year.

 Overall percentages of timely hip-fracture surgery in Piedmont in the 16 weeks before and after the national quarantine are illustrated in Figure 2, while a visual representation of the ITSA conducted on weekly percentages of surgical care is provided in Figure 3.

 It is clear by both charts that Piedmont did not experience any raise or drop in the amount of surgery since the second week of March 2020. As also confirmed by the regression coefficients in Table 2, pre-post differences in trajectories and levels of timely surgery did not differ between the study and control time series. Results were virtually unchanged after the exclusion of secondary diagnoses of hip fracture (Supplementary file 1, Figures S1 and S2).

**Table 2:** Regression Table of ITSA on Weekly Sex-, Age- and Comorbidity-Standardized Percentage of Surgery for Hip Fracture in Piedmont in the 16 Weeks Before and After Italy’s COVID-19 National Quarantine

**Variable**	**Coefficient (95% CI)**	***P *** **Value**
Surgery within 2 days		
Intercept	69.70 (63.28, 76.13)	<.001
*T*_t_	–0.18 (–0.84, 0.48)	.582
*X*_t_	0.27 (–4.97, 5.50)	.919
*X*_t_ *T*_t_	0.29 (–0.42, 1.01)	.413
*Z*	–2.75 (–10.36, 4.87)	.473
*ZT*_t_	0.41 (–0.50, 1.31)	.372
*ZX*_t_	–5.44 (–15.50, 4.61)	.283
*ZX*_t_ *T*_t_	–0.37 (–1.46, 0.72)	.503
Surgery within 1 day		
Intercept	44.72 (39.57, 49.88)	<.001
*T*_t_	–0.06 (–0.56, 0.45)	.820
*X*_t_	–3.59 (–8.70, 1.52)	.164
*X*_t_ *T*_t_	0.46 (–0.16, 1.09)	.145
*Z*	–1.19 (–8.29, 5.92)	.739
*ZT*_t_	0.20 (–0.62, 1.03)	.621
*ZX*_t_	3.84 (–8.64, 16.33)	.540
*ZX*_t_ *T*_t_	–0.68 (–1.93, 0.56)	.273
Surgery on the same day		
Intercept	16.44 (13.89, 18.98)	<.001
*T*_t_	–0.44 (–0.65, –0.23)	<.001
*X*_t_	2.45 (–0.38, 5.27)	.088
*X*_t_ *T*_t_	0.63 (0.25, 1.01)	.002
*Z*	–2.54 (–5.99, 0.91)	.145
*ZT*_t_	0.32 (–0.04, 0.67)	.078
*ZX*_t_	–4.14 (–9.07, 0.79)	.098
*ZX*_t_ *T*_t_	–0.33 (–0.91, 0.25)	.264

Abbreviations: ITSA, interrupted time-series analysis; CI, confidence interval; COVID-19, coronavirus disease 2019. *Notes:* Data observed the year before (2018/2019) are used for comparison. *T*_t _is time since the start of the study (November 20), *X*_t_ is an indicator variable that equals 1 in the weeks 11 to 26 of the tropical year (March 11, 2020/March 12, 2019 to June 30, 2020/July 1, 2019), and Z is an indicator variable that equals 1 in the “experimental” time series (November 20, 2019 to June 30, 2020). The post-quarantine trend between March 11, 2020 and June 30, 2020 can be obtained as *β*(*T*_t_) + *β*(*ZT*_t_) + *β*(*X*_t_*T*_t_) + *β*(*ZX*_t_*T*_t_).

## Discussion

 This study shows that in Piedmont, northern Italy, the percentages of hip-fracture surgery were unchanged during the lockdown period, without any differences among the proportion of patients operated on the day of admission, the following day and two days after admission. This is a significant result, as the percentage of patients treated within two days has been shown to be above the Italian average (53.2%) and close to the European Union average.^19^

 This result may be due to the reorganization of the healthcare system. Since the outbreak of the pandemic in February 2020, Italy’s healthcare system necessarily rearranged all its medical services in order to face the new emergency, which put acute-care structures, especially hospitals and emergency rooms, under a high amount of stress and work overload.^20-22^ In the field of orthopedics, many hospitals of northern Italy suspended non-urgent outpatient activities and cancelled elective surgery^13^; however, because acute trauma surgery could not be delayed, hospital services were reorganized with a redefinition of the roles of the various hospitals and the construction of clusters of hospitals. This new organization in Piedmont was based on the organizational model defined for polytrauma.^23^

 In Piedmont, the organization is managed by an operative team that has the task of evaluating the data from the cluster hospitals on a daily basis in terms of availability of resources and patients to be treated, suggesting different organizational solutions depending on the situation. In this reorganization, reducing as much as possible the length of hospital stay was a crucial point, in order to guarantee appropriate and timely care for patients needing immediate surgical interventions, and to maximize the availability of beds.^23^

 The importance of the role played by the reorganization of Piedmont is supported by another result. We found an increased concentration of admissions to research and teaching hospitals, together with a decrease in the relative number of admissions to the hospitals of local healthcare authorities and to private facilities. In order to allocate hospital paths completely dedicated to COVID-19 patients, separately from those of non-COVID-19 patients, smaller and local hospitals were partly converted into COVID-19 hospitals. We can infer that in converted hospitals the reduction of resources in terms of healthcare workers and beds ensured that the majority of patients were treated in bigger and best-equipped hospitals that were able to guarantee appropriate care even during the emergency.

 The positive result of timely hip-fracture surgery suggests a resilient response to the pandemic emergency. The new organization of orthopedic trauma surgery required a high degree of adaptability, making the system more efficient and changing the allocation of resources to avoid barriers in the access to health services during the pandemic. For example, in case of negative availability of operating rooms or healthcare workers (surgeons, nurses, etc) in the admitting hospital, alternative solutions have been adopted, including patient transfer, surgical team displacement or instrumentation displacement^23^. According to the World Health Organization (WHO), the capacity to adapting healthcare delivery within the system falls within the more traditional realm of resilience, defined as the ability to prepare for, manage and learn from shocks.^24^

 A resilient response is also indicated, albeit informally, by the ability to manage a significant increase in the hospitalization rate after a drop at the beginning of the pandemic period, with no apparent influence on the quality of care provided. This tentative result is coherent with a previous study showing that, together with an increase in surgical volumes, there is also an increase in the rate of hip fractures treated within two days of admission.^25^ This study suggests that, when dealing with high volumes of patients, health systems and hospitals change their organization, deploying smart processes and managing healthcare workers in order to achieve organizational effectiveness, which is defined as the organization ability to achieve the outcomes that the organization intends to produce.^26-29^

 The steady increase in the hospitalization rate, probably due to an increase in outdoor traumas following the progressive removal of restrictions in May 2020,^30^ seems to have enhanced and accelerated the ability to remodel the logistical and organizational management of trauma, with progressive reactivation of hospital beds and greater availability of operating rooms, a further definition of patient discharge procedures and a more efficient use of healthcare workers.

 The decrease that we observed in the number of hospitalizations for hip fracture after the declaration of Italy’s national quarantine is in contrast with the results of two studies conducted in Spain^31^ and in the United Kingdom,^32^ which showed that the hospital admission rate for patients with hip fracture was unchanged during the COVID-19 lockdown. On the other hand, our findings are similar to the results of another study carried out in Sardinia, Italy, which showed a general decrease in emergency room trauma visits by –60% during the lockdown period, possibly explicable by a decrease in the incidence of trauma as a consequence of the lockdown, which determined a significant reduction of mobility and outdoor activities.^33^ However, hip fractures typically involve elderly patients, which normally spend a significant amount of time at home, and are generally highly painful and severe.^34^ Hence, it is unlikely that patients with this kind of trauma decided not to go to the hospital due to fear of the ongoing pandemic. We could hypothesize that in Piedmont, which was one of the regions of Italy and Europe most stricken by COVID-19 in the first phase of the pandemic, with a proportion of older persons of 15%, people paid particular attention trying not to run into preventable domestic trauma in order to avoid the necessity to seek medical attention in a period of critical emergency. Extended confinement times, leading to reduced physical activity and sedentary behaviors, may then have made patients less active and frailer, and exposed patients to a greater risk of fracture in the post-lockdown period.^35,36^

 The findings of the present study have multiple policy implications. We focused on timely hip-fracture surgery as an indicator of the quality of care in a pandemic period. Indeed, with the onset of the COVID-19 pandemic, healthcare systems around the world had to face one primary challenge: how to effectively deliver adequate care to all patients, regardless of the entry point into the system, while protecting the well-being of non-COVID-19 patients and healthcare workforce. This study shows that it is possible to guarantee a high-quality and safe healthcare to patients for acute trauma, such as hip fracture, even in the most challenging working conditions. In addition, our findings bring to policy-makers’ attention the idea that quality measurement is essential during both times of stability and crisis. Data necessary to assess the quality and safety of care are crucial during pandemic periods, where healthcare processes rapidly change. Hence, improving data quality can significantly help clinicians strengthen care delivery in this difficult time and avoid a decrease of healthcare systems performances, which would have a negative effect on health outcomes.

 Secondly, our findings bring to policy-makers’ attention the role that resilience could play as an emerging property of the health system in the face of health emergencies. In this direction, the European Observatory on Health Systems and Policies has just published a new policy brief, “Strengthening the resilience of health systems,”^37^ which includes a framework to help policy-makers understand the strengths and the vulnerabilities of health systems and how to respond resiliently to system shocks. In this direction also some publications^38-40^ that, showing how resilience can enable organizations to cope with and recover from unexpected developments, invite policy-makers understand how it works and which factors facilitate or impede it into specific healthcare systems, in order to be adequately prepared for future threats.

###  Strengths and Limitations

 The main strength of this study is that it deals with an issue that is common to all public healthcare systems, as they are called to guarantee healthcare quality and safety, especially during a pandemic crisis. Furthermore, it focused on timely hip-fracture surgery, which is an important indicator adopted by international organizations (OECD, Eurostat, WHO) to measure quality of care. The main weakness of the study is that it was conducted on a specific region of a single country. Hence, further research in other countries is necessary to evaluate how the pandemic has impacted the quality of care in different contexts and to draw useful lessons on how to repeat this performance during future emergencies. On the other hand, our findings could be helpful to other European countries that share similarities with Piedmont in their healthcare systems and population health profiles.^41^ A second limitation, which is common to all studies based on administrative data, is that time between hospital admission and surgery could only be quantified in calendar days, which leads to the inclusion of a proportion of patients treated after 48 hours in the numerator counts. Moreover, despite accounting for individual-level confounding differences in age, sex and comorbidities to evaluate the outcomes of interest at the population level, we lacked relevant information on clinical features, clinical severity and socioeconomic factors, among the others. Other limitations are common to all studies based on healthcare administrative data, including lack of accuracy and differences in the coding criteria over time as well as across individuals and institutions. However, there is no reason to believe that such potential source of information bias might have significantly affected our difference-in-differences estimates. Lastly, we did not analyze relevant outcome indicators, such as mortality and readmission following hip fracture, that would provide stronger evidence in favor (or against) the healthcare resilience that our data on processes of care (timely surgery) and structural characteristics (hospital volumes) seem to suggest.

## Conclusion

 In Piedmont, northern Italy, the percentages of hip-fracture surgery were unchanged during the lockdown period, without any differences among the proportion of patients operated on the day of admission, the following day and two days after admission. This result suggests that healthcare systems, even during a global pandemic, can be resilient and able to get reorganized to guarantee a high-quality and safe healthcare to hip-fracture patients.

## Acknowledgements

 We would like to thank Roberta ONORATI of the Regional Public Health Observatory (SEPI), Local Health Unit TO3, Via Sabaudia 164, 10095, Grugliasco (To), Italy, for data extraction and sharing.

## Ethical issues

 This retrospective study was carried out in conformity with the regulations on data management with the Italian law on privacy (Legislation Decree 196/2003 amended by Legislation Decree 101/2018). Data were anonymized prior to the analysis at the regional statistical office of Piedmont, and each patient was assigned a unique identifier that eliminates the ability to trace the patient’s identity or other sensitive data. Anonymized administrative data can be used without a specific written informed consent and without the approval of an ethical committee when patient information is collected for healthcare management and healthcare quality evaluation and improvement (according to art. 110 on medical and biomedical and epidemiological research, Legislation Decree 101/2018).

 Patients and the public were not involved in the design or planning of the study. All procedures performed in this study were in accordance with the 1964 Helsinki Declaration and its later amendments.

## Competing interests

 Authors declare that they have no competing interests.

## Authors’ contributions

 Conception and design: MMG. Acquisition of data: MMG. Analysis and interpretation of data: JL, MMG, SR. Drafting of the manuscript: JL, MMG, and SR. Critical revision of the manuscript for important intellectual content: JL, MMG, SR, and MPF. Statistical analysis: JL. Supervision: MMG, JL, and SR.

## Disclaimer

 This paper represents only the opinions of the authors, and is the product of professional research.

## Supplementary files

Supplementary file 1 contains Table S1, Figure S1 and Figure S2.
