# Supplementary material for: Impact of COVID-19 on Timing of Hip-Fracture Surgeries: An Interrupted Time-Series Analysis of the Pre/Post-Quarantine Period in Northern Italy
Source: Int J Health Policy Manag. 2021 Sep 1;11(10):2083–9. doi: 10.34172/ijhpm.2021.103 (PMC9808284; doi:10.34172/ijhpm.2021.103)
Supplement: Supplementary file 1 — contains Table S1, Figure S1 and Figure S2. [file ijhpm-11-2083-s001.pdf]

Article title: Impact of COVID-19 on Timing of Hip-Fracture Surgeries: An Interrupted Time-Series Analysis of the Pre/Post-Quarantine Period in Northern Italy

Journal name: International Journal of Health Policy and Management (IJHPM)

Authors' information: Jacopo Lenzi[1], Stefano Rousset[2]*, Maria Pia Fantini[1], Maria Michela Gianino[2]

[1]Department of Biomedical and Neuromotor Sciences, Alma Mater Studiorum – Università di Bologna, Bologna, Italy.

[2]Department of Public Health Sciences and Paediatrics, Università di Torino, Torino, Italy.

(*Corresponding author: stefano.rousset@unito.it)

Supplementary file 1

Table S1. Patient- and hospital-level characteristics of the control and study groups, by 16-week period. Values are counts (percentages).

Characteristic	All	Control cohort (2018/19)		Study cohort (2019/20)		*P*-value
		Nov-20 to Mar-11	Mar-12 to Jul-01	Nov-20 to Mar-10	Mar-11 to June-30	
Sex						0.716
Male	2051 (25.1)	572 (25.5)	531 (25.7)	524 (24.4)	424 (24.6)	
Female	6135 (74.9)	1671 (74.5)	1539 (74.3)	1627 (75.6)	1298 (75.4)	
Age group, y						0.962
<80	2102 (25.7)	576 (25.7)	540 (26.1)	541 (25.2)	445 (25.8)	
80–84	1956 (23.9)	541 (24.1)	486 (23.5)	509 (23.7)	420 (24.4)	
85–89	2294 (28.0)	634 (28.3)	563 (27.2)	611 (28.4)	486 (28.2)	
≥90	1834 (22.4)	492 (21.9)	481 (23.2)	490 (22.8)	371 (21.5)	
Charlson index score						0.852
0	6963 (85.1)	1903 (84.8)	1751 (84.6)	1841 (85.6)	1468 (85.2)	
1	890 (10.9)	242 (10.8)	228 (11.0)	233 (10.8)	187 (10.9)	
≥2	333 (4.1)	98 (4.4)	91 (4.4)	77 (3.6)	67 (3.9)	
Hospital status/ownership						<0.001
LHA	5936 (72.5)	1651 (73.6)	1496 (72.3)	1603 (74.5)	1186 (68.9)	
Research/teaching	2054 (25.1)	532 (23.7)	506 (24.4)	502 (23.3)	514 (29.8)	
Private	196 (2.4)	60 (2.7)	68 (3.3)	46 (2.1)	22 (1.3)	
Hospital location						<0.001
Cities	3440 (42.0)	911 (40.6)	844 (40.8)	870 (40.4)	815 (47.3)	
Towns/suburbs	4745 (58.0)	1331 (59.3)	1226 (59.2)	1281 (59.6)	907 (52.7)	
Rural areas	1 (0.0)	1 (0.0)	0 (0.0)	0 (0.0)	0 (0.0)	
Hospital volume, HFs/y						<0.001
<50	38 (0.5)	22 (1.0)	3 (0.1)	7 (0.3)	6 (0.3)	
50–149	1106 (13.5)	309 (13.8)	295 (14.3)	296 (13.8)	206 (12.0)	
150–249	3887 (47.5)	1058 (47.2)	1026 (49.6)	1062 (49.4)	741 (43.0)	
≥250	3155 (38.5)	854 (38.1)	746 (36.0)	786 (36.5)	769 (44.7)	

Note: P-values were obtained with the chi-squared test.
Abbreviations: LHA, local healthcare authority; *HF*, hip fracture.

Figure S1. Sex-, age- and comorbidity-standardized percentages of hip-fracture surgery initiated within two days, within one day and on the same day as hospital admission in Piedmont in the 16 weeks before and after Italy's COVID-19 national quarantine. Secondary diagnoses of upper femur fracture are excluded (n = 198).

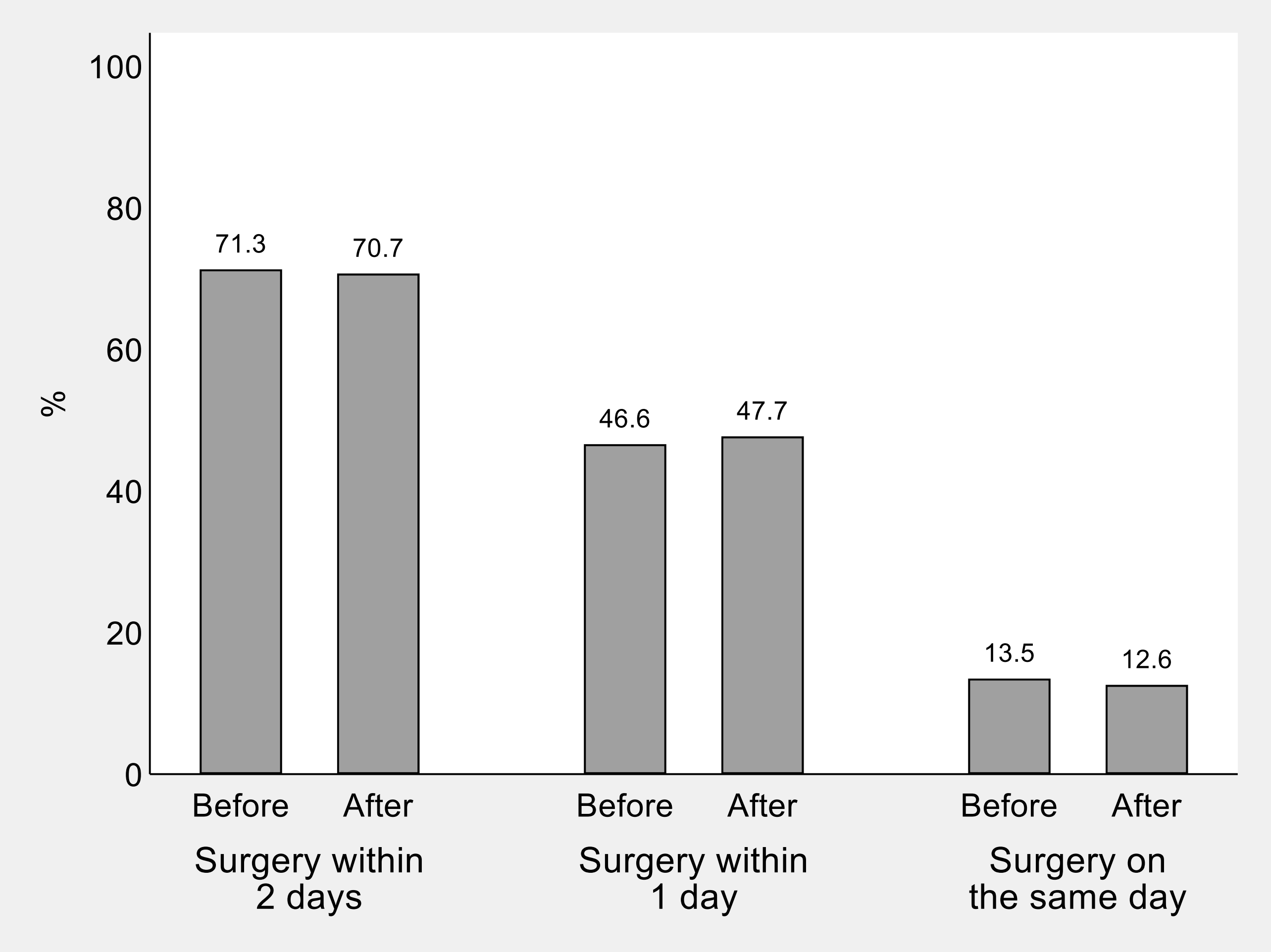

Note: No significant differences were found between the pre- and post-quarantine period on multivariable logistic regression analysis (two days: P-value = 0.707; one day: P-value = 0.586; same day: P-value = 0.463).

Figure S2. Interrupted time-series analysis of weekly sex-, age- and comorbidity-standardized percentages of hip-fracture surgery in Piedmont in the 16 weeks before and after Italy's COVID-19 national quarantine (dashed vertical line); data observed the year before (2018/19) are used for comparison. Secondary diagnoses of upper femur fracture are excluded (*n* = 198).

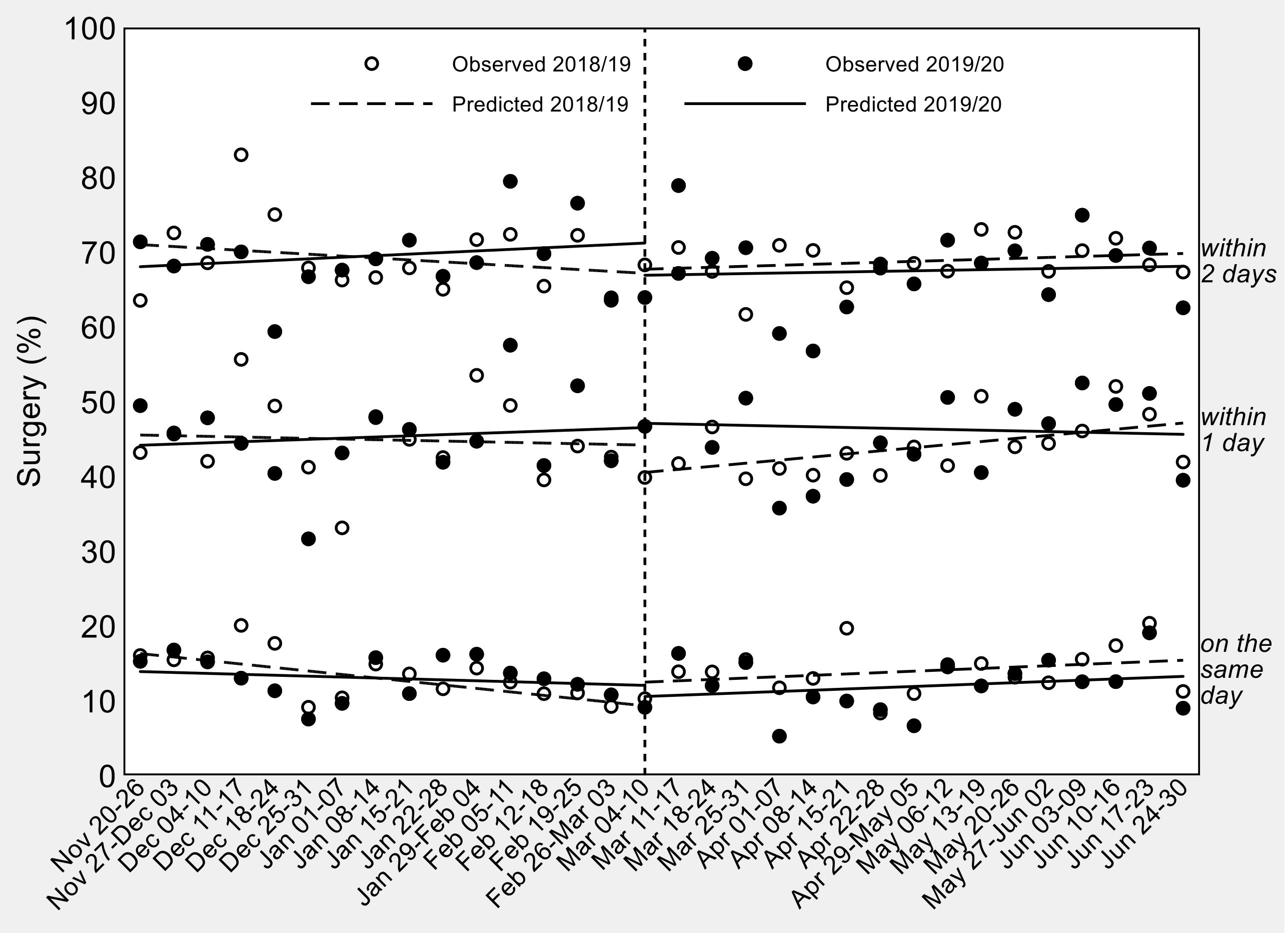

Note: The last day of the control period is July 1, because 2019 is a common (non-leap) year.
